# From bench to bedside a comprehensive review of pancreatic cancer immunotherapy

**DOI:** 10.1186/s40425-016-0119-z

**Published:** 2016-03-15

**Authors:** Paul R. Kunk, Todd W. Bauer, Craig L. Slingluff, Osama E. Rahma

**Affiliations:** Department of Medicine, Division of Hematology-Oncology, University of Virginia Health System, UVA Box 800716, Charlottesville, VA 22908 USA; Department of Surgery, Division of Hepatobiliary Surgery, University of Virginia Health System, Charlottesville, VA USA; Department of Surgery, Division of Surgical Oncology, University of Virginia Health System, Charlottesville, VA USA

**Keywords:** Pancreatic cancer, Immunotherapy, Cancer vaccine, Immune checkpoint, Tumor Immunology

## Abstract

The incidence of pancreatic cancer has been increasing while its 5-year survival rate has not changed in decades. In the era of personalized medicine, immunotherapy has emerged as a promising treatment modality in a variety of malignancies, including pancreatic cancer. This review will discuss the unique pancreatic tumor microenvironment, including the cells and receptors that transform the pancreas from its normal architecture into a complex mix of suppressor immune cells and dense extracellular matrix that allows for the unrestricted growth of cancer cells. Next, we will highlight the recently completed immunotherapy clinical trials in pancreatic cancer. Finally, we will explore the on-going immunotherapy clinical trials and future directions of this engaging and changing field.

## Background

Despite intensive research efforts to better understand its tumor microenvironment, the prognosis of pancreatic cancer remains dismal [1, 2]. The Pancreatic Cancer Action Network estimates that deaths from pancreatic cancer will be second only to lung cancer by 2020 [3]. Accordingly, novel treatment strategies for pancreatic cancer are desperately needed.

Immunotherapy is one of these novel strategies that has been under investigation in a variety of cancers. This review will focus on pancreatic cancer from an immune perspective, describing its immune microenvironment and the completed and ongoing clinical trials in this area.

## Review

### Pancreatic cancer from an immune perspective

Pancreatic cancer is unique from an immunological perspective. First, intratumoral effector T-cells are rare, in contrast to many other solid tumors for which infiltration of effector T-cells is often prominent [4, 5]. Second, the *RAS* oncogene drives an inflammatory program that establishes immune privilege in the pancreatic tumor microenvironment (PTME) [6]. Third, pancreatic cancer is associated with a massive infiltration of immunosuppressive leukocytes into the tumor microenvironment [4, 5]. Fourth, the development of pancreatic cancer is associated with a strong desmoplastic reaction that consists of multiple cell types, molecular factors, and extracellular matrix [7]. This dense desmoplastic stromal reaction is one of the hallmarks of pancreatic cancer and plays a vital role in promoting angiogenesis while evading from immune cells [4, 8, 9]. Studies have uncovered a rich communication between stellate cells (fibroblasts) and cancer cells [4, 8, 9]. The abundance of PDGF (platelet derived growth factor), fibronectin, proteoglycans and hyaluronic acid distorts the normal pancreatic architecture and transforms it into a complex, abnormal configuration of seemingly impenetrable walls [7]. Accordingly, this extensive stroma is not only a passive barrier for the immune system but rather interacts with cancer cells and participates in its progression and invasion [7].

**Fig. 1:**
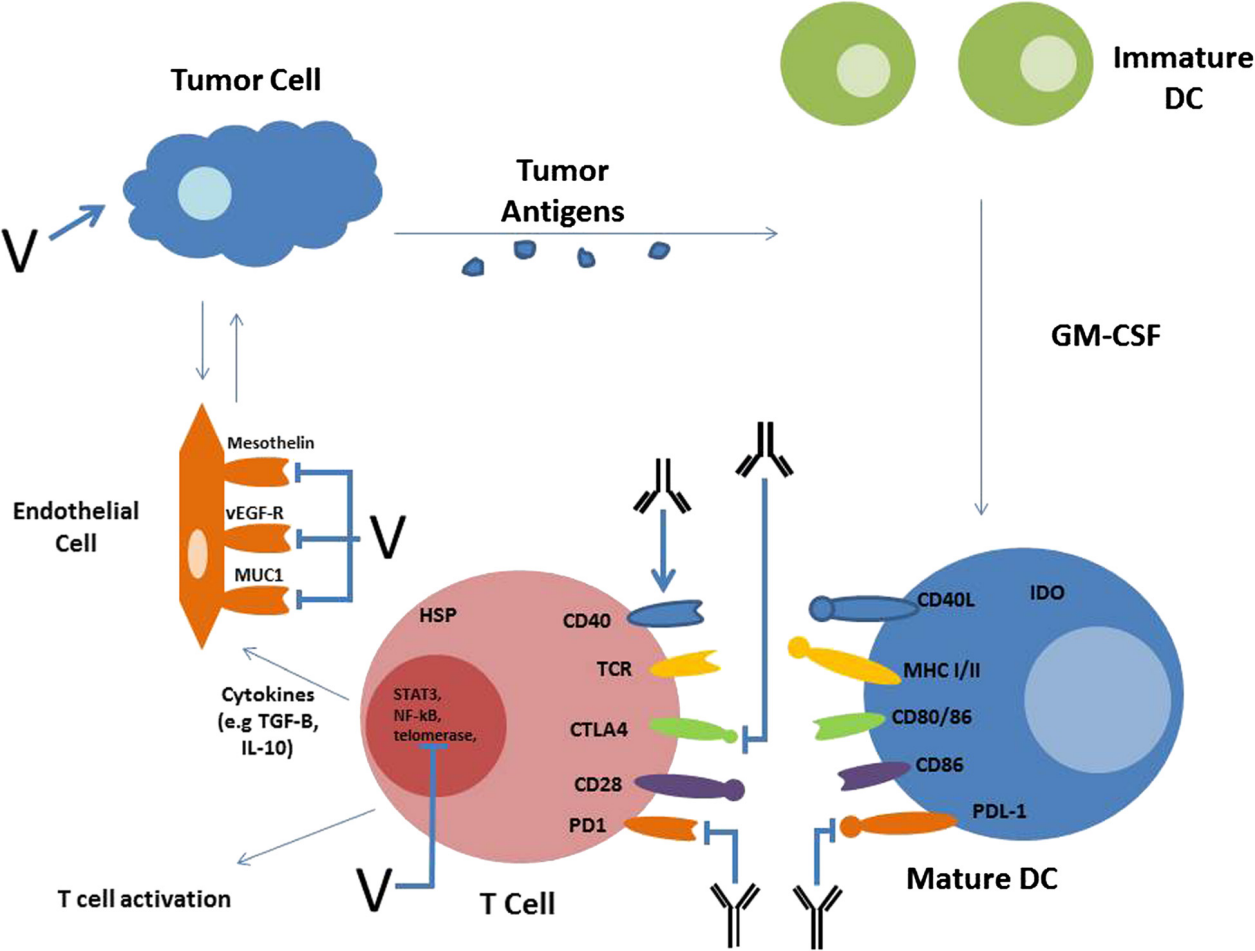
Pancreatic Cancer with Targeted Immunotherapy. DC (dendritic cell), GM-CSF (granulocyte macrophage colony stimulating factor), PD-1 (programmed cell death-1), PDL-1 (programmed cell death ligand-1), CTLA-4 (Cytotoxic T-lymphocyte associated protein-4), HSP (heat shock protein), TCR (T-cell receptor), MHC (major histocompatibility complex), vEGF-R (vascular epithelial growth factor-receptor), IDO (Indoleamine 2,3-dioxygenase), TGF (tumor growth factor), IL (interleukin), CD (cell differentiation), V (vaccine)

It is useful to examine the immune cells and receptors in pancreatic cancer based on their role in the development of an immune response and their correlation with prognosis. There are two immunological processes that determine the immune response against cancer cells: the effector process and the suppressor process. These cell types are summarized in Table 1 and Fig. 1.

**Table 1:** Cellular Microenvironment of Pancreatic Cancer

Cell	Role in pancreatic cancer	Relationship to outcome	Reference
NK	deactivated	↑tumor stage and ↓survival	[11–13]
CD8+ T-Cell	deactivated	↑tumor stage and ↓survival	[5, 8, 13]
CD4+ Th1-Cell	↓	↑tumor stage and ↓survival	[12–14]
TAM (M1)	↓	↑tumor stage	[4, 12–14]
DC	deactivated	↑survival	[8, 13, 17, 18]
MDSC	↑	↓survival	[5, 12, 16, 24]
Mast Cell	↑	↑metastases	[9, 13]
T-regs	↑	↓survival	[4, 5, 12, 22, 23]
TAM (M2)	↑	↑stage and ↓survival	[4, 12–14]
Fibroblast	↑	↑stage and ↓survival	[12, 13, 25, 27]
CD4 + Th2 Cell	↑	↑stage and ↓ survival	[5, 9, 12–14, 16, 25]

↑ increase, ↓ decrease, *DC* dendritic cell, *MDSC* myeloid derived suppressor cell, *NK* natural killer cell, *TAM* tumor associated macrophage

#### Effector immune cells

##### Natural killer cells (NK)

An increased number of NK cells have been shown to be associated with a better prognosis in a small set of 13 patients with pancreatic cancer [10], presumably due to their role in recognition and elimination of cancer cells. However, NK cells are typically found in a limited number in pancreatic cancer and often in a deactivated form due to the lack of NKG2D, a cell surface receptor found to be upregulated in activated NK cells [11–13].

##### CD8 Cytotoxic and CD4 helper T-cells or tumor infiltrating lymphocytes (TILs)

The presence of TILs in pancreatic cancer has been well described and it may represent the most important element in PTME [8, 9, 14, 5]. Among these TILs, the memory (CD45RO) CD8 T-cells are thought to be the major anti-tumor effector cells and their density in resected pancreatic tumors was found to correlate with survival [8, 9]. On the other hand, the role of CD4 T-cells is more complex. The Th1, the effector form, activates antigen presenting cells (APC) such as dendritic cells (DCs) while the ineffective form (Th2) plays a major role in tumor tolerance [13, 5]. Although the presence of both CD8 and CD4 T-cells correlates with a better prognosis [4, 8, 15], they are found in small numbers in the PTME, possibly due to effect of stroma and suppressor immune cells [15]. In addition, the number of CD8 effector T-cells decreases during the malignant transformation of pre-cancer cells [5, 8]. Studies have shown that Th1 cells are found in disproportionately lower concentrations among pancreatic cancer cells than Th2, suggesting an ineffective immune response against tumor cells [5, 16].

##### Dendritic cells (DCs)

The presence of DCs in the PTIM is essential in order to generate an anti-tumor immune response and, similar to TILs, is associated with a better prognosis in pancreatic cancer [8, 17, 18]. However, DCs are present in low numbers in the PTME and often in an immature form [8, 17, 18], thus likely limiting their ability to present foreign antigens to T-cells.

#### Co-stimulatory receptors and ligands

##### CD40

Is a co-stimulatory molecule that is expressed on T-cells and binds to its ligand (CD40L) on APCs, leading to the activation of lymphocytes [13]. In a retrospective analysis of patients with pancreatic cancer, Unek et al., showed that the expression of CD40 in pancreatic cancer tissue samples correlates with a trend towards improved progression-free survival (PFS) and overall survival (OS) [19]. Accordingly, CD40 represents a promising target in pancreatic cancer as described below.

##### OX-40 (tumor necrosis factor receptor superfamily member 4)

Is a member of the TNF receptor family found on T- cells and acts as a secondary co-stimulatory molecule as it requires other co-stimulatory molecules to be expressed first prior to its activation. The expression of OX-40 is found in high concentrations on activated T-cells. In pancreatic cancer increased levels of OX-40 was reported to correlate with better survival but this data needs to be validated on a larger scale [10].

##### 4-1BB (tumor necrosis factor receptor superfamily member 9)

Is also a member of the TNF receptor family and found on T-cells and NK cells. Upon interaction with its ligand 4-1BBL, it promotes T cell activation, particularly CD8 lymphocyte. 4-1BB also acts as a secondary co-stimulatory molecule, similar to OX-40 [20, 21]. However, there is currently no data available regarding the role of 4-1BB in the PTIM.

#### Suppressor immune cells

##### Tumor associated macrophages (TAMs)

These cells express the program cell death ligand (PD-L1) which is involved in immune suppression and T-cell apoptosis as described below. In PTIM, the presence of TAMs and it is association with poor outcomes and more frequent metastases has been well described [4, 13].

##### T-regulatory cells (CD4 + CD25 + FoxP3+) (T-regs)

T-regs are other subsets of TILs known for their immunosuppressive activity through the release of cytokines including TGF-β and IL-10. Based on few retrospective analyses, pancreatic tumors with low numbers of T-regs were found to have a significantly better survival compared to tumors with high numbers [4, 22, 23].

##### Myeloid derived suppressor Cell (MDSCs)

In pancreatic cancer, these cells were found in pre-malignant growths and increased in concentration as cancer cells grow suggesting a direct role in immune suppression and unrestricted cancer cell growth [5, 12, 16, 24]. Interestingly, high concentration of MDSCs in the peripheral blood was associated with poor outcomes in patients with pancreatic cancer [16]. Whether this correlation exists in the PTIM remains to be determined.

##### Fibroblasts/pancreatic stellate cell

Fibroblasts respond to a variety of molecules including CXCL12 (C-X-C motif chemokine 12) and produce VEGF (vascular endothelial growth factor) to stimulate angiogenesis in response to hypoxia or inflammation [7, 12, 13, 25]. In pancreatic cancer, the production of VEGF by fibroblasts is associated with cancer growth and worse prognosis [26, 27]. Therefore, the role of fibroblasts in the PTME is currently under intense investigation.

##### Mast cells

The role of mast cells in cancer has not been well-defined [9, 28, 29]. In pancreatic cancer, it has been suggested that low concentration of mast cells in the PTME correlates with increased survival [28] while increased concentration is associated with increased lymph node metastases based on retrospective analyses in small subsets of patients [29].

#### Co-Inhibitory Receptors and Ligands

##### Program-death (PD-1)

Is an inhibitory receptor that belongs to the B7-receptor family and interacts with its ligand PD-L1 (B7-H1) to down regulate signals by T-cells, leading to the induction of apoptosis in activated T-cells [13]. PD-1 is expressed on progenitor T cells, activated T- and B-lymphocytes, NK cells, and myeloid cells [13]. Patients with pancreatic cancer with PD-L1 positive tumors have a significantly worse prognosis than patients with PD-L1 negative tumors [27]. The PD-1/PD-L1 pathway is currently one of the most targeted pathways in cancer.

##### Cytotoxic T-lymphocyte associated protein-4 (CTLA-4)

Is a co-inhibitory molecule found on T- lymphocytes that deactivates these cells to induce apoptosis in response to interaction with APCs. This negative feedback loop is a key to normal immune function to prevent over-stimulation of T-cells and damage to healthy cells but is stimulated inappropriately in pancreatic cancer to create a microenvironment that promotes cancer growth rather than its recognition. CTLA-4 is overexpressed in pancreatic cancer cells and its overexpression was found to correlate negatively with survival in patients who underwent surgical resection [30].

##### CXCL12 (C-X-C motif chemokine ligand 12)

Is a chemokine that is found in high concentration in pancreatic cancer and is involved in fibroblast migration and proliferation. The increased concentration of CXCL12 in the cancer microenvironment creates a network of dense stroma restricting immune cells migration and recognition of cancer antigens. Feig et al., showed that the inhibition of this chemokine resulted in increased T-cell infiltration into pancreatic cancer in murine models [27].

##### T-cell immunoglobulin and mucin-domain containing molecule 3 (TIM-3)

Is a transmembrane protein that is involved in the regulation of Th1 lymphocytes. The interaction between TIM-3 and CD4 Th1 cells has been studied in pancreatic cancer patients and found to correlate with tumor vascular invasion [31]. However, the role of TIM-3 needs to be further characterized in pancreatic cancer and other malignancies.

##### Soluble lymphocyte activation gene-3 (LAG-3)

Is an important T-cell regulator that interacts with MHC class II molecules expressed on APCs. This interaction promotes activation and maturation of DCs but negatively regulates effector T-cells. LAG-3 has been shown to be necessary for T-regulatory cell activity and represents a novel target for therapy in pancreatic cancer [32].

##### Indoleamine 2,3-dioxygenase (IDO)

Is an enzyme involved in cleaving tryptophan into kynurenine. Tryptophan is required for T-cell activation and kynurenine leads to T-regs differentiation and chemotaxis [33]. Increased levels of IDO, as seen in pancreatic cancer, creates a microenvironment devoid of effector T-cells but rich in immunosuppressive T-regs [34]. IDO inhibitors are currently under investigation in pancreatic cancer and other malignancies (NCT02048709 and NCT02077881).

##### Galectins (Gal-1, Gal-3 and Gal-9)

Are immune modulating glycoproteins that are overexpressed in pancreatic cancer cells and thought to be involved in T-cell homeostasis. These glycoproteins have been shown to promote immune suppression in pancreatic cancer by promoting Th2 and T-reg transformation, restricting DC maturation and stimulating stellate cells [35, 36]. Their association with survival in pancreatic cancer has been conflicting, with several studies showing increased concentration associated with decreased survival [37, 38], while others showed an association with improved outcomes [39].

##### B7-H3

Is a member of the B7 ligand family that can be induced in activated dendritic cells, monocytes, and T cells leading to decrease Th1 type response and cytokine production. B7-H3 expression was found to correlate with lymph node metastases and advanced pathologic stage in patients with pancreatic cancer [40]. The inhibition of B7-H3 increased CD8+ TILs and inhibited tumor growth in mice [40].

### Pancreatic cancer vaccines

Cancer vaccines aim to stimulate the immune system against tumor cells by generating humoral and/or cellular immune responses. Many forms of cancer vaccines exist but generally they can be divided into synthetic and cellular-based vaccines.

#### Synthetic vaccines (summarized in Table 2)

**Table 2:** Completed immunotherapy clinical trials

Treatment type	Target	*N*	Additional therapy	Cancer stage	Immunologic response	Clinical outcome	Ref
Peptide vaccines	CEA	23	None	Resected or Metastatic	↑ IFN-γ T cell response by ELISPOT with increasing vaccine dose	37 % survival at 32 months	[77]
	CEA + MUC1	20	None	Metastatic	NR	mOS of 7.3 ms	[78]
	Gastrin 17	154	None	Metastatic	74 % + ELISA	↑OS by 54 % vs placebo (*p* = 0.03)	[51]
	Gastrin 17	383	None	Metastatic	Correlation between anti-gastrin17 titers and OS	No benefit	[53]
	Gastrin 17	30	None	Metastatic	67 % + ELISA	↑OS (4 to 7.2 ms if + IR (*p* < 0.01)	[52]
	GVAX + Mesothelin	90	Cyclophosphamide	Metastatic	NR	↑OS (4 to 6.2 ms)	[63]
	Hedgehog	59	Gemcitabine	Metastatic	NR	mOS 10 ms	[79]
	KRAS	23	None	Resected	85 % + DTH	10 year OS of 20 %	[56]
	KRAS	48	GM-CSF	Resected(10) and Metastatic (38)	58 % + DTH	↑OS (2 to 5.4 ms if + IR (*p* = 0.0002)	[54]
	KRAS	24	GM-CSF	Resected	11 % + DTH	mOS 20.3 ms	[55]
	KRAS	39	Gemcitabine	Resected	47 % + ELISpot	↑OS by 21.7 wks if + IR (*p* < 0.01)	[57]
	MUC1	16	SB-AS adjuvant	Resected	31 % + DHT	No benefit	[42]
	MUC1	6	Incomplete Freund's	Metastatic	17 % + ELISA	No benefit	[41]
	Telomerase	1062	Gemcitabine	Metastatic	NR	No benefit	[47]
	Telomerase	520	Gemcitabine	Metastatic	NR	No benefit	[48]
	Telomerase	48	GM-CSF	Metastatic	63 % + DHT	mOS of 4.3 ms if + IR (*p* < 0.01)	[50]
	Telomerase	178	Gemcitabine	Metastatic	NR	No benefit	[49]
	Trop-2	7	None	Metastatic	NR	No benefit	[80]
	VEGF	607	Erlotinib + Gemcitabine	Metastatic	NR	No benefit	[44]
	VEGF	535	Gemcitabine	Unresectable	NR	No benefit alone	[46]
	VEGF	150	Gemcitabine	Unresectable	NR	No benefit	[46]
	Wilm's Tumor gene-1	32	Gemcitabine	Unresectable	58 % + DTH	↑mOS by 7 ms if DHT + (*p* < 0.01)	[81]
Autologous: DC	MUC-1	49	Gemcitabine	Metastatic	↓65 % T-regs	2 CR, 5 PR ,10 SD	[59]
	MUC1	17	None	Resected and Unresectable	NR	mOS of 9 ms	[82]
	MUC-1	20	none	Metastatic	Correlation between CD38+ cells and OS	1 pt had remission of lung mets, 5 had stable disease. mOS 9.8 mos	[58]
	MUC-1	10	None	Resected	No difference	30 % OS 4 years	[83]
	MUC-1	2	None	Metastatic	↑117 % CD8+ MUC-1 specific cells	No benefit	[84]
	Wilm's Tumor gene-1	10	Gemcitabine	Metastatic	57 % + DTH	↑OS if + DTH	[85]
Allogeneic	GM-CSF	60	5-FU	Resected	↑ mesothelin + ELISPOT	↑OS (53 % to 76 % if + IR)	[61]
	GM-CSF	14	Adjuvant CRT	Resected	21 % + DTH	DFS of 25 ms if + R	[60]
	GM-CSF	30	Cyclophosphamide	Metastatic	↑Mesothelin ELISPOT	No benefit	[62]
Adoptive cell transfer	Mesothelin	6	None	Metastatic	NR	33 % with stable disease	[65]
	MUC1	28	None	Resected (20) and Unresectable (8)	↑10 % effector T-cells , ↓5.7 % Tregs	mOS 5 ms in unresectable, 19 % 3 year OS in resectable	[64]
	MUC1	20	None	Unresected	↑CD8+ T-cells	mOS 9.8 ms, 1 year OS 20 %	[58]
Immune checkpoint inhibitor	CD40	21	Gemcitabine	Unresected (7) and Metastatic (20)	N/A	↑mOS by 1.7 ms vs gemcitabine alone, 1 patient had complete resolution of hepatic metasteses	[68]
	CTLA-4	30	GVAX	Unresectable or metastatic	N/A	↑1 year OS by 20 % compared to GVAX alone	[75]
	CTLA-4	27	none	Unresected (7) and Metastatic (20)	N/A	1 patient delayed regression of hepatic metasteses	[70]
	PD-L1	14	none	Metastatic	N/A	No benefit	[69]

↑ increase, ↓ decrease, *CR* complete response, *CRT* chemoradiation, *CTLA-4* Cytotoxic T-lymphocyte associated protein-4, *DC* dendritic cell, *DTH* delayed typed hypersensitivity, *GM-CSF* granulocyte macrophage colony stimulating factor, *MDSC* myeloid derived suppressor cell, *mOS* median overall survival, *Ms* months, *N/A* not applicable, *NK* natural killer cell, *NR* not reported, *OS* overall survival, *PD-L1*, programmed cell death ligand-1, *PR* partial response, *R* response, *SD* stable disease, *TAM* tumor associated macrophages, *Wk* weeks

Synthetic vaccines are typically made from whole protein or peptides that match a pre-determined antigen to induce a T- cell response. Despite multiple large trials targeting MUC1 [41–43], VEGF [44–46], telomerase [47–50] and gastrin-17 [51–53], none have shown a meaningful survival benefit. These trials, however, were able to show significant immune responses to the targeted antigens. Some trials using mutated RAS peptide vaccine alone [54–56] or in combination with gemcitabine [57] have shown clinical benefit. Importantly, this benefit was mainly seen in patients who demonstrated positive immune responses [54, 56, 57].

#### Cellular-based vaccines (summarized in Table 2)

Cellular-based vaccines use cancer cells (either whole cells or cell lysates) as the source of the antigens, allowing the immune system to utilize multiple antigens rather than a single epitope. Overall the results of these trials are encouraging; however, each included a small number of patients making it difficult for meaningful interpretation. Cellular based vaccines can be divided into autologous or allogeneic vaccines based on the source of the cells.

##### Autologous vaccines

In this process, the patient’s own dendritic cells are isolated and pulsed with a specific antigen before being re-infused back to the patient. Multiple studies have used this vaccination approach targeting variety of antigens. MUC1 is one of the most targeted antigens given its overexpression in pancreatic cancer and its association with tumor invasion and metastasis. The clinical outcome of this vaccination method was not impressive; however, an interesting correlation with immune biomarkers was identified such as increased CD38 (a marker for activated lymphocytes) [58] and decreased T-regs [59].

##### Allogeneic vaccine

In this method of vaccination, a pancreatic cancer cell line is stimulated, usually with GM-CSF, in order to elicit an immune response when administered to patients with pancreatic cancer. A group at John Hopkins used this form of cancer vaccine (GVAX) in combination with 5-FU or chemoradiation in the adjuvant setting and showed an increase in PFS and OS in patients who developed an immune response against mesothelin [60, 61]. The same group also investigated GVAX in the metastatic setting in combination with low dose cyclophosphamide, to deplete T-regs, demonstrating an increase in mesothelin specific T-cell response with no survival benefit [62]. More recently, GVAX was combined with a Listeria vaccine that expresses mesothelin in 90 patients with metastatic disease. The combination of these 2 vaccines showed an improved OS compared to the allogeneic vaccine alone (6.1 vs 3.9 months, *p* = 0.01). Interestingly, patients who derived the most benefit of this combination were patients who received over 3 doses of the vaccine (9.7 vs 4.6 months, *p* = 0.01) and patients who received at least 2 previous chemotherapy regimens (5.1 vs 3.7 months, *p* = 0.001) [63]. This combination has currently a breakthrough designation by FDA while being investigated in patients with metastatic disease in a large ongoing clinical trial.

### Adoptive T- cell transfer (summarized in Table 2)

In this approach the patient’s T-cells are expanded and activated *ex vivo* then re-infused back to the patient. Based on the source and the method used for T-cell activation, adoptive T- cell transfer could be classified into: Tumor infiltrating lymphocytes (TILs), engineered T- cells that express a specific cancer T-cell receptor (TCR), and T- cells that express a chimeric antigen receptor (CAR). This methodology of immunotherapy had gained a lot of attention recently due to promising clinical outcomes in hematological malignancies. However, the efficacy of adoptive T- cell transfer remains to be determined in solid tumors including pancreatic cancer. Kawaoka et al., investigated MUC1-specific cytotoxic T lymphocytes (CTLs) in 28 patients and showed 19 % 3-year survival in patients with resectable disease along with increased effector lymphocytes and decreased T-regs [64]. More recently, the University of Pennsylvania group presented their experience using autologous T- cells modified with a chimeric antigen receptor (CAR) that recognizes mesothelin in pancreatic cancer patients with refractory metastatic disease. Of the 6 patients treated 2 had stable disease with one patient had a decreased PET avidity of hepatic metastases. Overall, the treatment was well tolerated [65]. The carcinoembryonic antigen (CEA) is another attractive target in pancreatic cancer that is currently been explored in clinical trials (NCT01723306, NCT00004178 and NCT01212887). New generations of CAR therapies are under investigation with a focus on increasing their activities and specificities and decreasing their toxicities. The CAR T-cells efficacy could be enhanced by engineering the intracellular domain to contain co-stimulatory molecules such as 41BB and OX40 or combining CAR T-cells therapy with immune modulators such as cyclophosphamide in order to deplete T-regulatory cells (NCT02465983) or immune checkpoint inhibitors such as CTLA-4 and anti-PD1 antibodies.

### Immune checkpoint inhibitors and co-stimulatory agonists (summarized in Table 2)

Immune checkpoint inhibitors represent a paradigm shift in cancer treatment due to their promising clinical activities in melanoma and other malignancies [66, 67]. However, a limited number of studies targeting these immune checkpoints have been completed in pancreatic cancer. The first study to investigate the co-stimulatory agonists in pancreatic cancer used CD40 agonist in combination with gemcitabine in 21 patients with locally advanced or metastatic disease. This study demonstrated promising outcomes with an improvement of median OS compared to gemcitabine alone and one patient having a complete resolution of his liver metastases [68]. In the area of immune checkpoint inhibitors, both CTLA-4 and PD-L1 inhibitors were investigated in patients with locally advanced or metastatic pancreatic cancer in 2 clinical trials. The clinical outcomes were disappointing, although, only small number of patients were treated on both trials [69, 70]. To date, the only immune checkpoint inhibitor to show activity in pancreatic cancer is MEDI4736 (anti-PD-L1), which showed a PR rate of 8 % in a preliminary analysis of this going trial [71]. While this suggests a response can be achieved with single immunotherapy, it remains dismal and maybe improved by combination therapy. This approach is currently being investigated in few ongoing trials as detailed in Table 3.

**Table 3:** On-going immunotherapy clinical trials

Treatment type	Phase	Target	*N*	Additional therapy	Stage	Identifier
Chimeric antibody	I,II	Ensituximab	116	None	Metastatic	NCT01040000^a^
DNA vaccine	I	VEGFR-2	72	None	Metastatic	NCT01486329^a^
Fungal vector vaccine	II	RAS	176	Gemcitabine	Resected	NCT00300950^a^
Viral vector vaccine	I	Small Pox Virus	36	None	Metastatic	NCT00574977^a^
Allogeneic vaccine	I	CEA	48	GM-CSF	Metastatic	NCT00028496^a^
	I	Donor Lymphocyte	37	None	Metastatic	NCT00161187^a^
	I	Dendritic cells	12	Poly-ICLC	Unresectable	NCT01677962
	II	GM-CSF	60	Cetuximab + Cyclophosphamide	Metastatic	NCT00305760^a^
	II	GVAX	56	None	Metastatic	NCT00389610
	II	GVAX	87	Cyclophosphamide	Resectable	NCT0072744
	II	GVAX	19	Cyclophosphamide, Radiation, FOLFIRINOX	Resected	NCT01595321
	II	GVAX +/- Mesothelin	93	Cyclophosphamide	Metastatic	NCT01417000
	II	GVAX +/- Mesothelin	240	Gemcitabine, Capecitabine, 5-FU, Irinotecan, Erlotinib or Cyclophosphamide	Metastatic	NCT02004262
	II	IFN-α + GM-CSF	14	Cyclophosphamide	Metastatic	NCT00002773^a^
	III	Virulizin	400	Gemcitabine +/- 5-FU	Metastatic	NCT00040092^a^
Autologous vaccine	I	CEA	24	None	Metastatic	NCT00004604^a^
	I	CEA	14	None	Metastatic	NCT00027534^a^
	I	CEA	24	Denileukin Diftitox	Metastatic	NCT00128622^a^
	II	CEA	48	IL-2	Metastatic	NCT01723306
	I	Dendritic Cells	2	Gemcitabine + Stereotactic Radiosurgery	Metastatic	NCT00547144^a^
	II	KLH	35	Radiation	Metastatic	NCT00868114
Immunotherapy	I	B7-H3	93	none	All	NCT01391143
	I	CD40	10	Gemcitabine + nab-paclitaxel	Metastatic	NCT02588443
	I	CD40	10	Gemcitabine	resected	NCT01456585^a^
	II	CTLA-4	82	None	Metastatic	NCT00112580^a^
	I	CTLA-4	37	Gemcitabine	Metastatic	NCT00556023^a^
	I	CTLA-4	28	Gemcitabine	Metastatic	NCT01473940
	II	CTLA-4 + GVAX	92	FOLFIRINOX	Metastatic	NCT01896869
	II	IDO	98	Gemcitabine + nab-paclitaxel	metastatic	NCT02077881
	I	IL-1-Ra	13	FOLFIRINOX	Metastatic	NCT02021422
	I/II	PD-1	56	Capecitabine + Radiation	Resectable and Borderline Resectable	NCT02305186
	I	PD-L1	1038	none	All	NCT01693562
Peptide vaccine	I,II	Alpha (1, 3) galactosyltransferase	7	None	Metastatic	NCT00255827^a^
	III	Alpha (1, 3) galactosyltransferase	280	FOLFIRINOX	Locally Advanced	NCT01836432
	III	Alpha (1,3) galactosyltransferase	722	Gemcitabine, 5-FU, radiation	Resected	NCT01072981
	I,II	CEA	28	None	Metastatic	NCT00529984^a^
	I	CEA + MUC1	18	None	Unresectable	NCT00669734
	I	hCG-β	36	None	Metastatic	NCT00648102^a^
	I	hCG-β	48	None	Metastatic	NCT00709462^a^
	I	Heat Shock Protein	16	None	Resected	NCT00003025^a^
	I/II	Hedgehog	122	Gemcitabine	Metastatic	NCT01130142^a^
	I	Hedgehog	21	FOLFIRINOX	Unresectable	NCT01383538
	I	MUC1	25	None	Resected or Locally Advanced	NCT00008099^a^
	I/II	MUC5AC	90	Gemcitabine + nab-paclitaxel	Unresectable	NCT01834235
	I	P53	12	None	Unresectable	NCT01191684^a^
	II	P53 + RAS	70	IL-2	Metastatic	NCT00019084^a^
	I	RAS	7	None	Metastatic	NCT00006387^a^
	I	RAS	33	None	Metastatic	NCT00019006^a^
	III	Telomerase	1110	Capecitabine + Gemcitabine	Metastatic	NCT00425360^a^
	I	TGF-β	168	Gemcitabine	Resectable and unresectable	NCT01373164
	I	Trophoblast glycoprotein	44	None	Metastatic	NCT00056537^a^
	I/II	VEGF	17	Gemcitabine	Unresectable	NCT00655785^a^
	I	VEGFR-2	21	Gemcitabine	Metastatic	NCT00622622^a^

*5-FU* 5-flurouracil, *CEA* Carcinoembryonic antigen, *CTLA-4* Cytotoxic T-lymphocyte associated protein-4, *FOLFIRINOX* folinic acid, fluorouracil, irinotecan, oxaliplatin, *GM-CSF* granulocyte macrophage colony stimulating factor, *hCG* Human chorionic gonadotropin, *IDO* indoleamine 2,3-dioxygenase, *IFN* interferon, *IL-2* interleukin-2, *KLH* keyhole limpet hemocyanin, *MUC* mucin, *N* number, *PD* programmed death, *PD-L* programmed death ligand, *Poly-ICLC* carboxymethylcellulose, polyinosinic-polycytidylic acid, and poly-L-lysine double-stranded RNA, *TGF* Transforming growth factor, *VEGF* Vascular endothelial growth factor, *VEGF-R* Vascular endothelial growth factor receptor

^a^Study listed as complete but results not published

### Combination therapy

Due to the lack of meaningful clinical benefits of cancer vaccines, the potential positive immunological effect of chemotherapy and radiation therapy, and the promising outcomes of immune checkpoint inhibitors, the focus has shifted towards combining these modalities. Gemcitabine, a standard chemotherapy that is used traditionally to treat pancreatic cancer, has been found to mediate immunological effects such as tumor associated antigen cross presentation by dendritic cells and the induction and expansion of cytotoxic T cells responses in addition to reduce the number of myeloid suppressor cells [72, 73]. Radiation therapy can also increase the immunogenic properties of tumor cells by enhancing MHC class I expression, thereby increasing their vulnerability to CTLs. Another frequent effect of DNA damage inflicted by radiotherapy or chemotherapy is the increase in the expression of death receptors (in particular Fas/CD95 and TNF-related apoptosis-inducing ligand [TRAIL] receptors, enabling lysis of the tumor cells by Fas/CD95 ligand and TRAIL-positive immune effectors [74]. As detailed in Table 3, the majority of ongoing trials investigate a combination strategy of the immunotherapy with chemotherapy, radiation or both. Of interest are multiple trials targeting mesothelin and/or GVAX with chemoradiation and multiple immune checkpoint inhibitors combined with chemotherapy. Our group is currently investigating the immunological effect of the combination of chemoradiation and anti-PD-1 as a neoadjuvant treatment in patients with resectable or borderline resectable pancreatic cancer compared to neoadjuvant chemoradiation alone (NCT02305186). This neoadjuvant setting will allow investigators to study the effect of combination therapy on the tumor microenvironment. Another promising combination by the Hopkins group combined GVAX with anti-CTLA-4 and demonstrated a 1-year improvement in OS by 20 % compared to GVAX and cyclophosphamide alone [75].

## Conclusion

Despite the ongoing efforts outlined in this review, the prognosis of pancreatic cancer remains dismal. With the recent progress in cancer immunotherapy, there are glimmers of hope in new immune targets with more being identified each year. These advancements are moving from the bench to the bedside at a rapid pace, with the hope of translating into improvements in clinical outcomes. We believe that immunotherapy represents a promising modality in pancreatic cancer. However, there still remains much to be learned about the pancreatic immune microenvironment and its role in the immune escape of cancer cells. In order to develop an active strategy to enhance the immune response against pancreatic cancer that could be translated to a promising clinical outcome we must focus our efforts on increasing the density of the intratumoral effector T-cells; decreasing or inhibiting the immunosuppressive cells and receptors; and understanding the role of the stromal reaction and its interaction with pancreatic cancer immune microenvironment. Indeed, the recent encouraging data of patients with mismatch-repair deficient colorectal cancer responding to pembrolizumab requires further investigation, particularly as it may be relevant for a small number of patients with pancreatic cancer [76]. Improved response rates and survival benefits may be achieved by using combination therapies; identifying novel biomarkers in order to select the group of patients who may drive the most benefit of cancer immunotherapy; and implementing novel clinical trials designs that allow for tumor samples collection in order to understand the mechanism of action and resistance of pancreatic cancer to immunotherapy.

## Abbreviations

↑: increase
↓: decrease
4-1BB: Tumor Necrosis Factor Receptor Superfamily Member 9
APC: antigen presenting cell
CD: cell differentiation
CR: complete response
CRT: chemoradiation
CTLA-4: Cytotoxic T-lymphocyte associated protein-4
CXCL12: C-X-C motif chemokine 12
DC: dendritic cell
DTH: delayed typed hypersensitivity
ELISA: enzyme linked immunosorbent assay
FOLFIRINOX: folinic acid, fluorouracil, irinotecan, oxaliplatin
Gal: Galectins
GM-CSF: granulocyte macrophage colony stimulating factor
HSP: heat shock protein
IDO: Indoleamine 2,3-dioxygenase
IFN-γ: interferon-gamma
IL: interleukin
LAG-3: Soluble Lymphocyte Activation Gene-3
MDSC: myeloid derived suppressor cell
MHC: major histocompatibility complex
mOS: median overall survival
ms: months
NA: not applicable
NK: natural killer cell
NR: not reported
OS: overall survival
OX-40: Tumor Necrosis Factor Receptor Superfamily Member 4
PD-1: programmed cell death-1
PDGF: platelet derived growth factor
PD-L1: programmed cell death-ligand 1
PR: partial response
PTME: pancreatic tumor microenvironment
R: response
SD: stable disease
TAM: tumor associated macrophages
TCR: T-cell receptor
TGF: tumor growth factor
TIL: tumor infiltrating lymphocyte
TIM-3: T-cell Immunoglobulin and mucin-domain containing molecule 3
vEGF: vascular epithelial growth factor
vEGF-R: vascular epithelial growth factor-receptor
